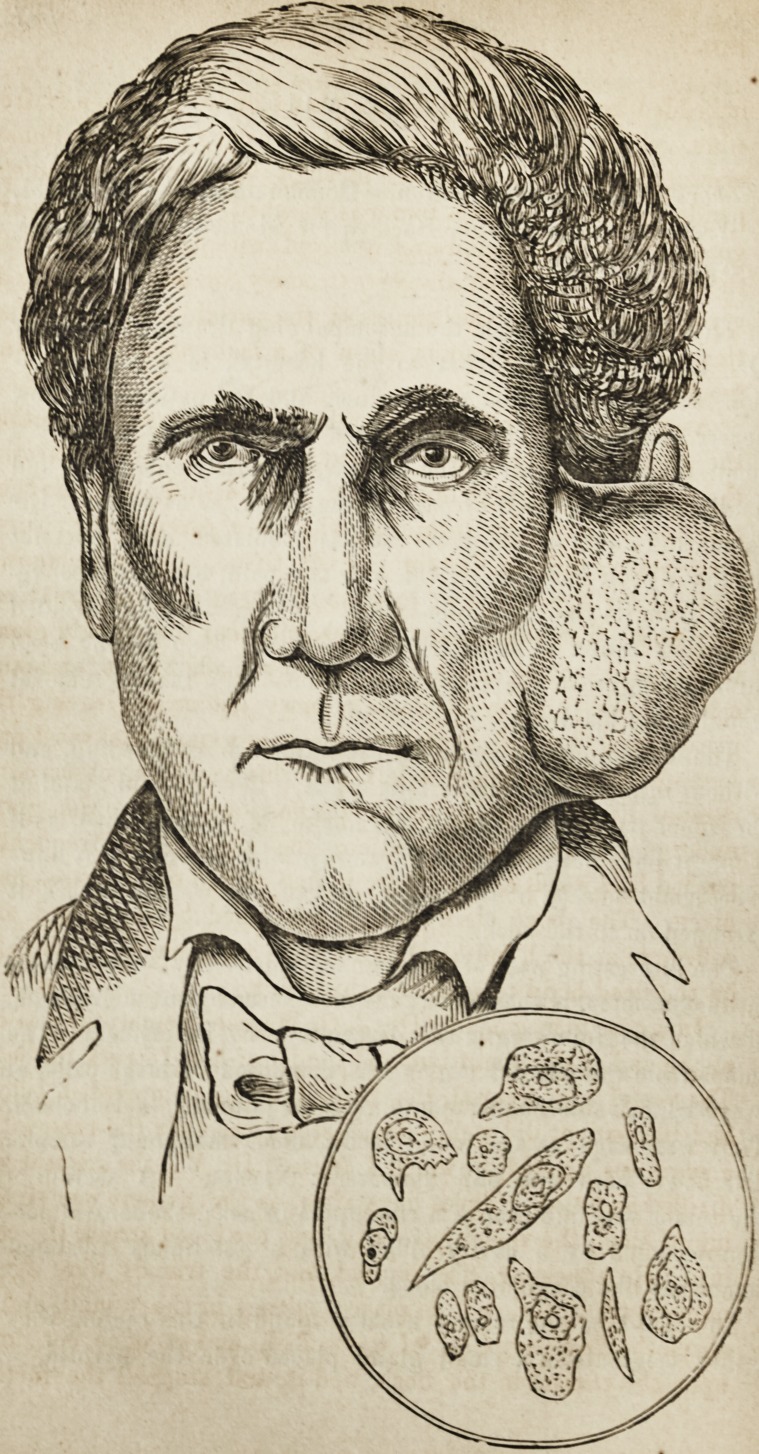# Tumors of the Parotid Region

**Published:** 1857-10

**Authors:** J. M. Warren

**Affiliations:** to the Boston Society for Medical Improvement, April 13, 1857.


### ARTICLE VIII.

*Tumors of the Parotid Region.* Communicated by Dr. J. M. Warren, to the Boston Society for Medical Improvement, April 13, 1857.

The tumor, of which the accompanying drawing, made by Dr. L. M. Sargent, artist to the hospital, is a representation, was removed three weeks since, and the patient has now nearly recovered. Its origin dated twenty years back by a small swelling in front of the left ear, gradually enlarging, and finally possessing itself of part of the cheek, so as to cover the ramus and angle of the jaw. It was lobulated, soft, but not fluctuating; it had never given him any pain, but was inconvenient from its size and appearance. The patient had avoided an earlier resort to an operation, probably from having been informed eighteen years ago by a medical man, that its removal would cause his death.

The dissection was accomplished without much bleeding, and without injury to any important organ. The parotid gland at the upper part of the tumor was not to be distinguished, as if it might have been removed by absorption; remains of it however, could be seen at the lower part of the wound, where it extended on to the neck.

The interesting part of the tumor, apparently so innocuous in its character, is the appearance discovered by the microscope, of which the following is the description by Dr. Ellis. "The tumor from the parotid region was composed of large cells, of every conceivable shape, containing large nuclei and nucleoli. They were such as are found in the most malignant tumors. The power used was 382 diameters; Nachet." A section of the tumor was like that of a ripe apple or pear, the central portion occupied by a cavity filled with a gelatinous substance somewhat like coagulated blood.

In regard to the tumors usually found in this region, they either originate in a small gland placed over the parotid, or imbedded in its substance, or are placed under its lower or free edge, which is expanded to form a coating over the tumor, making it necessary to dissect through that portion of the expanded gland before the tumor is reached. These tumors are generally innocuous, and are removed without great risk, although they are almost always extremely vascular. So far as my experience goes, the tumors of the parotid itself are affections of a serious character, often of a malignant nature, and generally scirrhous.

As to the practical question which is often raised, whether the gland can be removed without the ligature of the carotid, the result of my experience is this. The parotid gland has been removed by me in six instances, which are given below; three for scirrhous disease, one for erectile tissue, one for melanosis, and one for hypertrophy; in none of these was the great artery tied. The experiment of dissecting out the parotid gland in the dead subject has been frequently made by me, and with a little care this can be done in most instances, leaving the great vessels behind, although sometimes a small backward projecting bit of the gland is left, and this has been observed to escape disease. But in scirrhous affections, where the gland undergoes a gradual induration, the vessels are frequently pushed backward, as they were in one or two of the cases here given. The above observation is confirmed by my friend and colleague at the hospital, Dr. Gay, who made similar dissections on the dead body to ascertain this point.

In a case mentioned by Dr. J. C. Warren the carotid was cut at the end of the operation, and the jet of blood struck the wall. The vessel was secured, the carotid being compressed below, and the patient did well. In a second case for the removal of a scirrhous parotid, in which I assisted Dr. W., the carotid was divided and tied. Three days after, as the patient was straining at stool, the vessel gave way, and the blood struck the ceiling. He almost at once fainted, and the friends were fortunately sufficiently cool to place a sponge in the wound, and to check the flow partially. I was called, and at once cut down upon the carotid in the neck, tied it, and stopped the further effusion of blood. Bérard, in his monograph on this subject, mentions many instances of removal of this gland without ligature of the carotid.

The following cases, one of which has already been published in this Journal, are interesting as illustrative of the above facts, and also from some peculiarities in the nature of the tumors themselves.

Case I.—A young married woman entered the hospital in April, 1847, with a tumor of the parotid gland of one year's duration. Eight years previously she had a tumor below and behind the right ear, which was very hard and occasionally painful; the integument was not discolored. At the end of four years, having attained the size of a robin's egg, it was removed. The wound, she thinks, never cicatrized; and in four months, the tumor having re-appeared in the midst of the scar, was again removed. Its character was similar to the preceding, with the exception that the surface was nodulated. The wound healed as usual, but the cicatrix remained very red. The present tumor began to form about a year since in the same place, and is now as large as a pullet's egg, projecting an inch, with a surface nodulated and red. Commencing below the ear, it proceeds upward and forward to about half an inch in front of the meatus.

On the first day of May, the patient being etherized, the diseased mass was surrounded by an elliptical incision. From the situation of the disease the dissection was made very slowly, requiring nearly an hour for the operation. At the lower part was a firm adhesion to the fibres of the sterno-mastoid, a portion of which muscle was removed. At the upper part it was necessary to carry the dissection down to the articulation of the jaw, below and behind the angle of which the disease descended deeply, rendering necessary the exposure of the tendon of the digastricus. On raising the tumor to continue the deep dissection, violent efforts at vomiting, difficulty of breathing, and convulsive retchings from the traction exercised on the deep nerves came on, so that it was necessary to desist, and destroy the small portion of the base of the tumor with the hot iron.

A few ligatures were applied, and the wound, measuring three inches and a half vertically by two transversely, was covered by a wet cloth. The growth measured vertically three inches. The face was more or less paralyzed after the operation.

This lady was discharged from the hospital on the 18th of June, all the disease being apparently removed, and the whole wound reduced to a diameter of one-third of an inch. In the middle of September following a letter was received, saying that the patient remained well, and the wound was healed.

CASE II.—A robust, hearty-looking man belonging to the State of Maine, about 34 years old, consulted me for a tumor in the right parotid gland. Twenty years before a tumor had been removed from the same situation, which soon re-appeared as a small, hard tubercle under the ear. After remaining stationary fifteen years it increased, till at the time of his visit it had attained the size of a hen's egg, was of a bluish color, lobulated, and having a hard base surrounded by small cysts, pushing upward the lobe of the ear, and extending inward so as to involve the lower half of the parotid gland. Upon consultation, it was thought best to attempt its removal without ligature of the carotid.

The patient being under the influence of ether, the tumor was removed by a very slow and careful dissection; its base had undergone osseous degeneration, and involved the facial nerve, causing a paralysis of his face. In a week he was able to return home.

CASE III.—The patient was a farmer, 52 years old. Twenty-five years ago a tumor made its appearance in front of the ear. This imperceptibly increased, giving him no pain or inconvenience until two months since, when it was injured by a blow, and since then has rapidly increased in size. The night after the blow, he perceived that there was some insensibility in the skin in front of the tumor. For some time past he has been unable to close the right eye. "Now, there is an oval, prominent, even, well-defined tumor in front of the right ear, overlying the ramus of the lower jaw, and occupying the position of the parotid gland. Its long axis is parallel with a line drawn from the angle of the jaw to the external angle of the orbit. Its greatest length is three inches, width two inches. Upper margin is on a level with the angle of the eye; lower margin with the angle of the jaw; posterior is overlapped by external ear. Integument is movable; not discolored. Tumor is of firm consistence; not tender on pressure; not attached to bone, yet but slightly movable. Does not move with lower jaw; cannot be felt in mouth. There is much numbness of cheek in front, and a dull, but not severe pain in the tumor itself." *Hospital Record.*

When the patient entered the hospital, one or two glands in the neighborhood of the tumor were enlarged, apparently from the effect of some irritating application he had made for the purpose of discussing it. Under treatment, these, with one exception, disappeared. He was extremely desirous of having the tumor removed, and on a consultation of the surgeons it was decided that the attempt should be made.

The patient being etherized with chloric ether, an incision was made from just above the superior border of the tumor to a little below its inferior part. This was crossed by another incision commencing at the mastoid process, and terminating on the cheek. The fibrous capsule of the gland was now cut into, and the tumor gradually loosened by dissecting carefully around its circumference. Its adhesions were so close, and the texture so firm, that it was found impossible to proceed but with great caution; the vessels that were divided under the edges of the tumor being secured with much difficulty. The tumor was first loosened from its attachment to the zygomatic process, then dissected from the masseter muscle, the transverse facial artery and the parotid duct being cut away at this stage of the dissection. It was next detached from its firm adhesions to the sterno-mastoid muscle and mastoid process, and its adhesions to the ear cut off. Finally, by means of the blade and handle of the knife it was separated, from before backward, from the great artery and vein which lay imbedded in its posterior wall, the latter being cut and tied. Four or five arteries required ligatures. An enlarged gland in the neighborhood was removed separately from the tumor.

The mouth was found paralyzed after the operation. The eye, which the patient was unable to close before, either in sleep or when wide awake, was found, a few days subsequent to the removal of the tumor, to drop down so as partially to cover the eyeball when he was asleep.

An examination of the tumor, after its removal, showed it to be the parotid in a scirrhous state, the microscope disclosing an abundance of cancerous cells; with it was included a lymphatic gland imbedded in its lower and under portion.

The presence of the parotid duct and the facial nerve in the tumor now shown, together with its anatomical relations, left no doubt as to the organ diseased.

CASE IV.—*Melanotic Disease of the Parotic Gland.*—C. L., a seaman from Maine, unmarried, 25 years of age, entered the hospital in 1852 with a melanotic tumor. For three years previous he had had a small black fungus upon the right cheek in front of the ear, and about a year previous the glands of the neck became somewhat irritated.

At the time of his admission there was an irregular, lobular tumor, the upper part of which was surmounted by a black fungus as large as a walnut, occupying the right parotid region, where it was slightly movable, but descending below and behind the angle of the jaw, where it was immovable.

The patient being under full etherization, the tumor was surrounded by an elliptical incision, and the dissection commenced. Blood, however, followed every stroke of the knife, and poured from the whole surface of the tumor, so as only to be checked, and the further prosecution of the operation allowed, by applying the freezing mixture and constant compression of the carotid. After the removal of some easily detached portions, by the advice of the surgeons present, the operation was finally terminated by transfixing it at the base with a very strong double ligature, and tying it in two segments. Previously to this many ligatures were placed on bleeding vessels, and the hemorrhage was very large; in short, wherever the tumor was cut or broken, a great amount of thick granular fluid, of a jet black color, flowed out.

Upon partial recovery from the effects of the ether, hemorrhage from the tumor continued to such an extent as to render it necessary to again encircle the base by a strong ligature. The tumor ultimately returned.

Case V.—A married man from Nova Scotia, 58 years of age, entered the hospital in April, 1854, with a parotideal tumor of twenty-six years' standing. This tumor was situated on the left side, and came on without any known cause. It extended downward, lifting up the lobe of the ear, partially closing the meatus, and causing some deafness. The integument over it was injected, but not adherent. The pain for a short time had been severe, preventing sleep. It was considered of so formidable a character, that the surgeons to whom he had applied declined interfering with it.

The patient being etherized, the tumor was removed by a crucial incision through the skin, followed by a careful dissection, and was terminated without the ligature of the carotid artery. The hemorrhage was very free, and the dissection could only be prosecuted by stopping from time to time, and applying the freezing mixture, so as to allow an inspection of the parts to be divided. It was found to consist of hypertrophied glandular tissue.

In a short time he was discharged well, and when heard from on Nov. 6th, 1856, he was in good health.

Case VI.—Mrs. B., 37 years of age, applied to me in the month of November, 1853, with a tumor occupying the seat of the parotid gland. It had appeared first two years since in front of the ear, and in its increase had extended downward and under the ear, lifting up the lower part of that organ. It was a little movable, and did not project much from the surface beyond the surrounding parts. It appeared firmly attached below, was somewhat lobulated, and imparted a sense of elasticity to the touch. Her father died of cancer.

The tumor was exposed by a careful dissection, but on its investments being cut into, a granular matter like cancer exuded from it, and the hemorrhage was very violent, welling up as if from the carotid, or some very large vessel. It was, therefore, found necessary to terminate the operation by the ligature en masse, as in the case of the melanotic affection.

The disease, examined under the microscope by Dr. Shaw, exhibited well marked cancer-cells.

The subsequent history of the patient was not learned.

### EDITORIAL DEPARTMENT.

## Figures and Tables

**Figure f1:**